# Effects of Management, Dietary Intake, and Genotype on Rumen Morphology, Fermentation, and Microbiota, and on Meat Quality in Yaks and Cattle

**DOI:** 10.3389/fnut.2021.755255

**Published:** 2021-11-11

**Authors:** Changsheng Hu, Luming Ding, Cuixia Jiang, Chengfang Ma, Botao Liu, Donglin Li, Abraham Allan Degen

**Affiliations:** ^1^State Key Laboratory of Grassland Agro-Ecosystem, School of Life Sciences, Lanzhou University, Lanzhou, China; ^2^Qinghai Provincial Key Laboratory of Adaptive Management on Alpine Grassland, Qinghai University, Xining, China; ^3^Gansu Devotion Biotechnology Co., Ltd., Zhangye, China; ^4^Qinghai Qilian Yida Meat Co., Ltd., Qinghai, China; ^5^Desert Animal Adaptations and Husbandry, Wyler Department of Dryland Agriculture, Blaustein Institutes for Desert Research, Ben-Gurion University of the Negev, Beer Sheva, Israel

**Keywords:** grazing, feedlot, yak, cattle, rumen microbiota, meat quality

## Abstract

Traditionally, yaks graze only natural grassland, even in harsh winters. Meat from grazing yaks is considered very healthy; however, feedlot fattening, which includes concentrate, has been introduced. We questioned whether this change in management and diet would have an impact on the rumen and meat quality of yaks. This study examined the morphology, fermentation, and microbiota of the rumen and the quality of meat of three groups of bovines: (1) grazing yaks (GYs, 4-year olds), without dietary supplements; (2) yaks (FYs, 2.5-year olds) feedlot-fattened for 5 months after grazing natural pasture; and (3) feedlot-fattened cattle (FC, Simmental, 2-year olds). This design allowed us to determine the role of diet (with and without concentrate) and genotype (yaks vs. cattle) on variables measured. Ruminal papillae surface area was greater in the FYs than in the GYs (*P* = 0.02), and ruminal microbial diversity was greater but richness was lesser in the GYs than in the FC and FYs. Concentrations of ruminal volatile fatty acids were greater in the yaks than in the cattle. In addition, both yak groups had higher protein and lower fat contents in meat than the FC. Meat of GY had a lower n6:n3 ratio than FY and FC, and was the only group with a ratio below *r*, which is recommended for healthy food. Essential amino acids (EAA), as a proportion of total AA and of non-essential AA of yak meat, met WHO criteria for healthy food; whereas FC did not.

## Introduction

The yak (*Poephagus grunniens*) is the sole bovine species that has adapted well to the Qinghai-Tibetan plateau. This ruminant is crucial for the livelihood of residents, providing meat, milk, dung, and fiber. Traditionally, yaks grazed natural pasture all year, without supplementary feed, even in long, harsh winters. Because of the clean environment and year-round grazing of natural pasture, yak meat has been regarded as healthy and organic, and is in high demand (1). However, with increased number of yaks in recent years and the widespread degradation of grasslands, the natural pasture cannot sustain the stocking rate of yaks. Different options are being examined to raise yaks, and feedlots are being established. Weaned calves are being bought from herders and fattened on concentrate feed.

Dietary intake affects rumen fermentation and development (2), and meat properties. For example, supplementary concentrate increased rumen microbial abundance and enhanced epithelium development in Tibetan sheep (3), while a high forage diet increased intramuscular fat in steers (4). We questioned how feedlot fattening with concentrate feed would affect the rumen and meat quality of yaks. To answer this query, we examined rumen morphology, fermentation, and microbiota, and meat quality in yaks grazing only natural pasture, without supplements, yaks fattened with concentrate in a feedlot for 5 months after only grazing natural pasture for 2 years, and Simmental cattle fattened in a feedlot. We also questioned whether dietary intake (concentrate vs. no concentrate) or genotype (yaks vs. cattle) would have greater influence on the measurements.

## Materials and Methods

### Animals and Sample Collection

Three groups of bovines were included in the study: (1) four, 4-year-old male yaks that only grazed natural pasture with no supplements (GYs); (2) three 2.5-year-old male yaks that grazed natural pasture for 2 years but were fattened in a feedlot for the last 5 months (FYs); and (3) three 2-year-old male Simmental cattle that were fattened in a feedlot (FC). Simmental is the popular beef breed in the area and is raised only in feedlots. Attempts to graze this breed have been unsuccessful, because they are not adapted to harsh pasture conditions. The GYs and FYs were of the same genetic background and grazed in Qilian County (3,200 m. a. s. l.), Qinghai province; The FYs and FC were fattened in a feedlot in Zhangye city (2,000 m. a. s. l.), Gansu province, adjacent to Qilian County, on a total mixed ration (Table 1). Because the growing rate of GYs was slower than that of FYs and FC, they were slaughtered at an older age than the other two groups. The aim of the study was to follow the natural management system for yaks and Simmental cattle in the area, so that the results would be relevant for the meat sold to consumers. The grazing yaks are slaughtered at 4 years of age and older; whereas the feedlot yaks are fattened between 2 and 2.5 years and slaughtered at 2.5 years of age. We wanted the GYs and FYs to be from the same genetic background to eliminate genetic differences from the comparison between the yak groups. This was accomplished but also limited the number of yaks available for the study.

**Table 1:** Diet composition of feedlot-fattened yaks and feedlot-fattened cattle (DM basis).

**Ingredients**	**Content g/100 g**	
	**Yak**	**Cattle**
Corn silage	0	8
Oat hay	0	12
Wheat straw	20	0
Ground corn	47.2	47.2
Cottonseed meal	9.2	9.2
DDGS	4.96	4.96
Corn germ meal	4.24	4.24
Soybean hull	2.8	2.8
Spray corn husks	4.08	4.08
Soybean	3.6	3.6
MgO	0.24	0.24
Limestone	0.88	0.88
CaHPO_4_	1.2	1.2
NaHCO_3_	0.48	0.48
NaCl	0.96	0.96
Flavoring agent	0.016	0.016
Vitamins	0.016	0.016
Minerals	0.112	0.112
Plant oil	0.016	0.016

The FYs and FC were slaughtered in an abattoir on November 21 and 22, 2019, and the GYs on December 12, 2019, at 05:00 after fasting 18 h, but they had free access to water for up to 2 h before slaughter. Total rumen contents were collected immediately after slaughter, mixed thoroughly, which also dislodged much of the microbiota adhering to the solid particles, and strained through four layers of cheesecloth, as described by Hu et al. (5). Fifty ml rumen fluid was transferred immediately to the laboratory and stored at −80°C until analysis. Tissues (~1 cm^2^) were collected from the ventral region of the rumen. The left *muscularis longissimus* (12–13th rib levels) was collected from each yak, cooled for 24 h at 4°C, and then stored at −20°C until analysis.

### Ruminal Histology

The ruminal tissues were rinsed with physiological saline, fixed in 10% buffered formalin solution for 48 h, dehydrated in a graded series of absolute ethanol (30, 50, 70, and 90%), cleared with benzene, and then saturated with and embedded in paraffin wax. Blocks were cut into 5-μm sections using a rotary microtome (RM2235; Leica, Wetzlar, Germany), and sections were stained by hematoxylin-eosin (6). The morphology of the tissues was examined by microscopy and photographed (Smartzoom 5; Zeiss, Jena, Germany) and images were analyzed for length and width of papillae with the Motic imaging software (Motic, Kowloon, Hong Kong). The number of papillae was estimated following the method described by Shen (7). In brief, the number of papillae per cm^2^ of mucosa was counted by video camera imaging using the Motic imaging software (Motic, Kowloon, Hong Kong). The total surface area of papillae per cm^2^ was calculated as length × width × 2, times the number of papillae per cm^2^.

### Rumen Microbial DNA Extraction and Sequencing

The frozen rumen fluid was thawed on ice for DNA extraction. Microbial DNA was extracted with TIANamp Stool DNA Kit (Tiangen Biotech Co., Ltd, Beijing, China), following the protocol of the manufacturer. After checking the quality and quantity of DNA with a DNA spectrophotometer (ND-1000; Nano Drop, Wilmington, DE, United States), the DNA pellet was freeze-dried and dissolved in Tris-EDTA (TE) buffer (pH 8) containing DNase-free RNase (100 μg/ml), and stored at −20°C. The primers used were designed according to the conserved region, and the end of the primers was added with a sequencing connector, which was amplified by PCR. The products were purified, quantified, and homogenized to form a sequencing library. The targets in the V3-V4 region of the bacterial 16S rRNA gene were amplified using 338F (5′-ACTCCTACGGGAGGCAGCA-3′) and 806R (5′-GGACTACHVGGGTWTCTAAT-3′), as reported by Wei et al. (8). Phusion High-Fidelity PCR Master Mix and GC Buffer were used for PCR reaction. The PCR temperature regime was as follows: denaturing at 94°C for 1 min, annealing at 51°C for 1 min, and elongating at 72°C for 1.5 min. At the end of 30 cycles, a final extension was dwelled at 72°C for 8 min. The PCR products were purified by 2% agarose gel electrophoresis. Finally, the obtained amplicons were sequenced using an Illumina HiSeq platform to generate paired 250-bp reads.

Microbial diversity analysis was assessed with an Illumina HiSeq sequencing platform, using the paired-end (PE) sequencing method to construct a small fragment library for sequencing. The FLASH v1.2.7 software was used to splice the PE reads of each sample through overlap, and the resulting spliced sequence was the original Tags data (Raw Tags). The Trimmomatic v0.33 software filtered the Raw Tags obtained by splicing for Clean Tags, and the UCHIME v4.2 software identified and removed chimera sequences to obtain the final Effective Tags. UCLUST in the QIIME 1.9.1 software was used to cluster Tags at 97% similarity level, obtain operational taxonomic units (OTUs), and annotate the OTUs based on the Silva and UNITE taxonomy databases.

### Determination of Ruminal Volatile Fatty Acids

Ruminal volatile fatty acids (VFAs) were determined as described by Stewart and Duncan (9). In brief, after thawing at 4°C for 2 h, 2 ml of the rumen fluid was centrifuged at 4,830 × *g* at 4°C for 10 min, and then 0.15 ml metaphosphoric acid was added to 1.5 ml of supernatant and homogenized. The mixed solution was centrifuged at 5,670 × *g* at 4°C for 15 min, and the supernatant was used to determine the VFAs (acetate, propionate, and butyrate) using a gas chromatograph (GC, SP-3420A; Beijing Beifen-Ruili Analytical Instrument Co., Ltd., Beijing, China) equipped with an AT-FFAP type capillary column (30 m × 0.32 mm × 0.5 μm) and a flame ionization detector, with the temperature regime as: the column temperature was maintained at 90°C for 1 min, increased to 120°C at 10°C /min for 1 min, then increased from 120 to 150°C at 10°C/min, and maintained at 150°C for 3 min.

### Meat Composition

Meat samples were dried and milled (Wiley Mill No 1; Arthur Thomas Co, Philadelphia, PA, United States) through a 2-mm screen. Protein content was determined by the Dumas method (combusting samples, AACC method 46-30.01) using an elemental analyzer (Elementar Vario MACRO Cube; Elementar, Hanau, Germany), and water and intramuscular fat (IMF) contents were determined using a SMART Trac rapid fat analyzer (CEM Corporation, Matthews, NC, United States) (Methods 985.14 and 985.26, AOAC, 1990). Ash was determined by burning the samples at 550°C for 4 h in a furnace (TM-0912p; ICHCM, Beijing, China). Amino acids were determined by an amino acid analyzer (Biochrom 30+; Biochrom, Cambridge, United Kingdom). Meat fatty acid (FA) concentrations were determined as described by Sukhija et al. (10). Briefly, the total fat in a 0.2-g sample was extracted with 2 ml benzene containing an internal standard (0.5 mg/ml C19) and 3 ml of 5% methanolic HC1 (10 ml of acetyl chloride to 100 ml of anhydrous methanol) in culture tubes. The tube was vortexed for 1 min and heated for 2 h in a water bath at 70°C. After cooling to room temperature, 5 ml of 6% K_2_CO_3_ was added, and the tube was vortexed again for 0.5 min and centrifuged for 5 min at 75 × *g* at 4°C. The clear upper benzene layer was transferred to another tube for determination of fatty acids using a gas chromatograph mass spectrometer (GC-MS, QP2020, Shimadzu, Kyoto, Japan) fitted with a fused-silica capillary column (100 m × 0.25 mm × 0.2 μm, sp-2560; Supelco, Belfonte, PA, USA), following Ding et al. (11).

The atherogenic index (AI) and thrombogenicity index (TI) of meat were calculated based on the FA concentrations (12):

AI = (C12:0+4×C14:0+C16:0)/(MUFA + PUFA);

TI = (C14:0+C16:0+C18:0)/[(0.5 × MUFA) + (0.5 × n6 PUFA) + (3 × n3 PUFA) + ^*^n3/n6 PUFA]

where C12:0, C14:0, C16:0, and C18:0 are lauric, myritoleic, palmitoleic, and stearic acids; MUFA is monounsaturated fatty acids, and PUFA is polyunsaturated fatty acids (g/100 g total fatty acids).

### Statistical Analysis

Ruminal VFAs and meat chemical, fatty acid, and amino acid compositions were analyzed by one-way ANOVA (SPSS v26, Chicago, IL, United States). The microbial alpha diversity index, which included Shannon and Chao1 indices, was evaluated using the Mothur (version v.1.30) software. The relative abundance of rumen bacteria (phyla and genera), operational taxonomic units (OTUs), Kyoto Encyclopedia of Genes and Genomes (KEGGs), and principal coordinates analysis (PCoA) were determined using the vegan package in R 3.4.1.

Following log transformations, microbial data deviated from normality (Shapiro–Wilk's tests, *P* < 0.05). Non-parametric factorial Kruskal–Wallis sum-rank tests were performed to test for differences among groups at the bacterial phylum and genus levels, and Dunn's test (13) was performed to separate means where significance was found. Measurements are reported as their non-transformed values. Correlations among the top 10 most abundant bacterial genera with bovine genotype (FY vs. FC) and feed (GY vs. FY), were tested using Spearman rank correlation analysis of R software. Statistical significance was accepted at *P* < 0.05 and as a tendency at 0.05 ≤ *P* < 0.1. When significant differences were found, means were separated by Tukey's test.

## Results

### Rumen Histology

**Figure 1:**
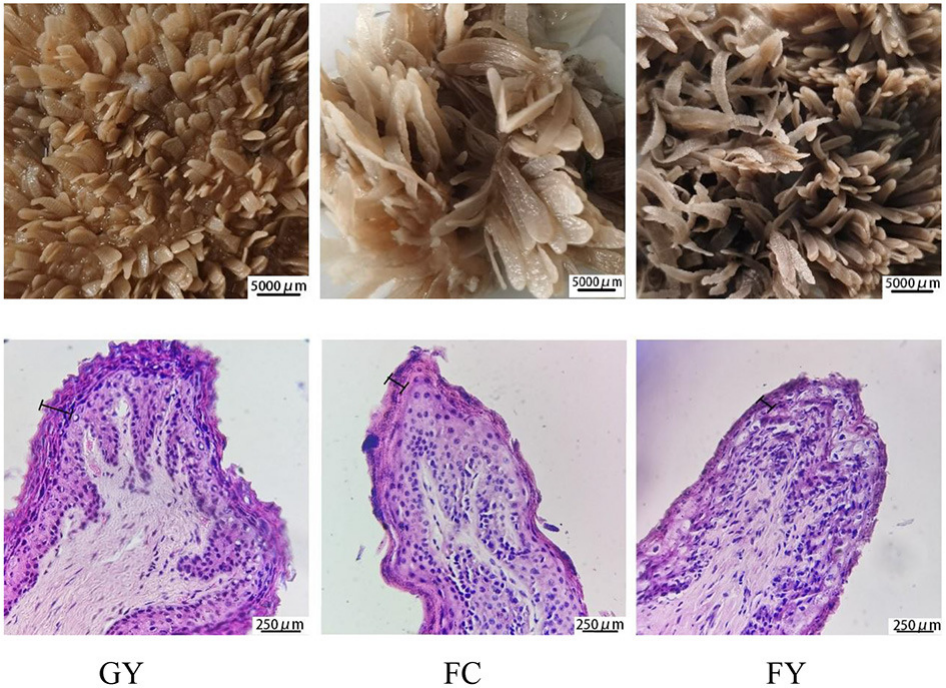
Morphology structure of rumen papillae of grazing yaks (GYs), feedlot-fattened cattle (FC), and feedlot-fattened yaks (FYs) (The 250-μm scale denotes stratum corneum thickness).

Diets affected the morphology of ruminal papillae of the three bovine groups. The papillae surface area in FY was larger than that in GY, but did not differ from that in FC. GY had a thicker *stratum corneum* and wider connective tissue of ruminal papilla (Figure 1, Table 2), and wider but shorter ruminal papillae than FY and FC (*P* < 0.05) (Table 2). Of the three groups, GY had the highest papillae density, while FC had the lowest.

**Table 2:** Rumen morphology of grazing yaks (GYs), feedlot-fattened cattle (FC), and feedlot-fattened yaks (FYs).

**Item**	**GY**	**FC**	**FY**	**SEM**	***P-*value**
*Stratum corneum* width,μm	129.5^b^	38.3^a^	38.0^a^	12.81	<0.01
Papillae width, mm	2.42^b^	1.60^a^	1.55^a^	0.15	0.012
Papillae length, mm	5.30^a^	10.25^b^	15.95^b^	1.64	0.018
Papillae density, n/cm^2^	30.67^b^	26.00^a^	47.33^c^	3.29	<0.001
Papillae surface area, mm^2^	25.63^a^	31.60^a^^,^^b^	45.62^b^	3.60	0.016

a,b *Means within a row followed by different lower-case letters differ significantly from each other (P < 0.05)*.

### Rumen Fermentation

Ruminal acetate and total volatile fatty acid concentrations were greater (*P* < 0.05) in GY and FY than in FC, but concentrations of ruminal propionate, butyrate, and valerate did not differ among the three bovine groups (Table 3).

**Table 3:** Rumen volatile fatty acids (VFAs) of GYs, FC, and FYs.

**Item**	**GY**	**FC**	**FY**	**SEM**	***P*-value**
Acetate, mM	21.93^b^	9.72^a^	19.63^b^	2.32	0.04
Propionate, mM	9.83	3.49	8.19	1.35	0.13
Butyrate, mM	5.13	2.82	7.51	1.03	0.19
Valerate, mM	0.42	0.73	1.08	0.12	0.25
TVFA, mM	37.9^b^	15.6^a^	36.5^b^	4.52	0.04
A/P	2.36	2.59	2.78	0.17	0.67

a,b *Means within a row followed by different lower case letters differ significantly from each other (P < 0.05)*.

*TVFAs, total volatile fatty acids; A/P, acetate/propionate*.

### Ruminal Microbiota

**Figure 2:**
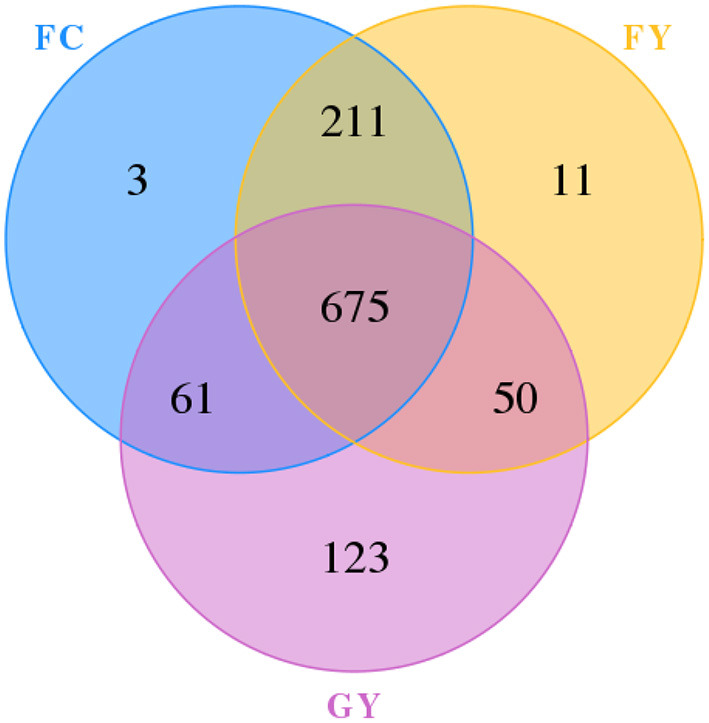
Ruminal operational taxonomic unit (OTU) of bacteria in GYs, FC, and FYs.

**Figure 3:**
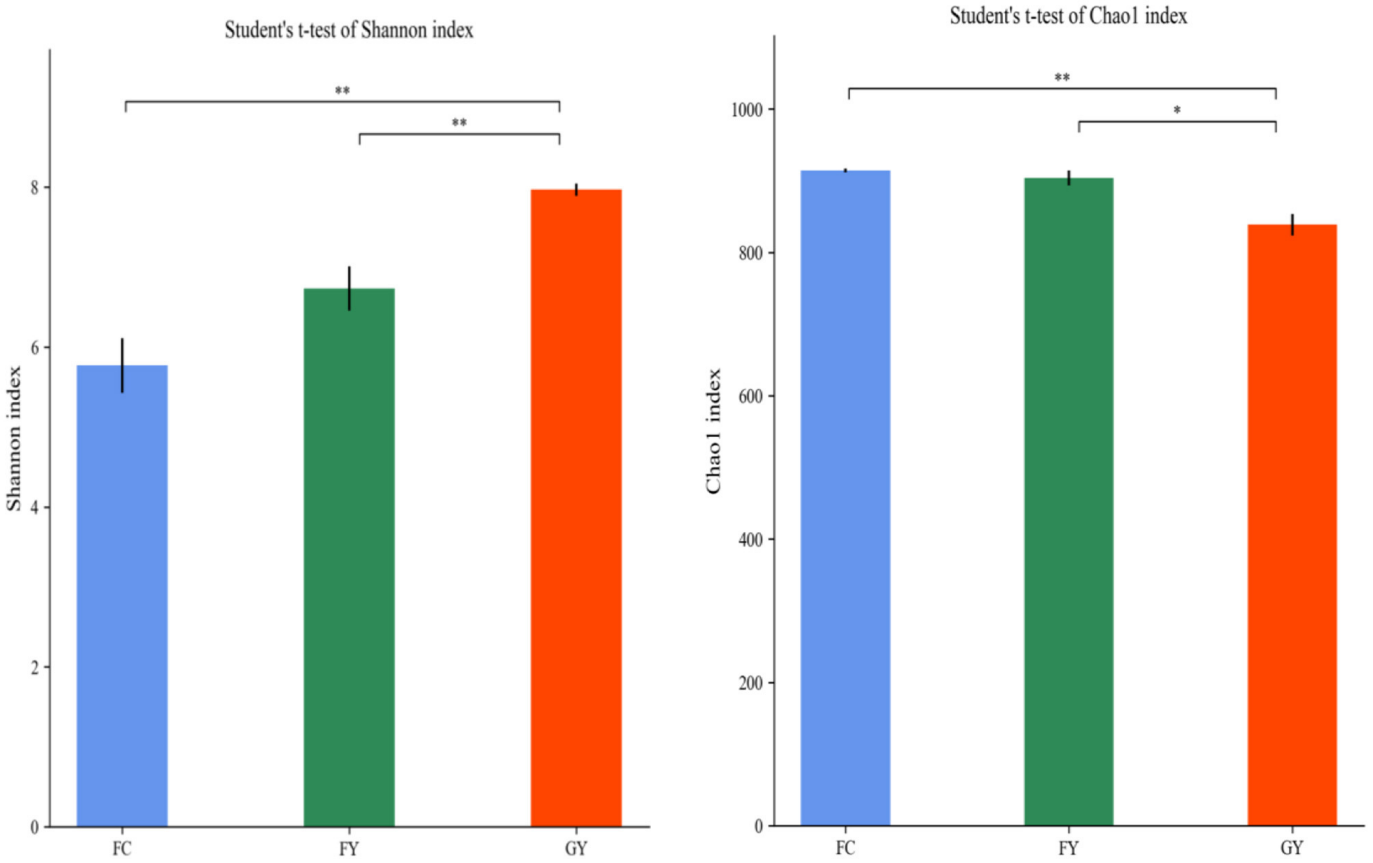
Ruminal bacteria diversity of GYs, FC, and FYs. *P < 0.05; **P < 0.01.

**Figure 4:**
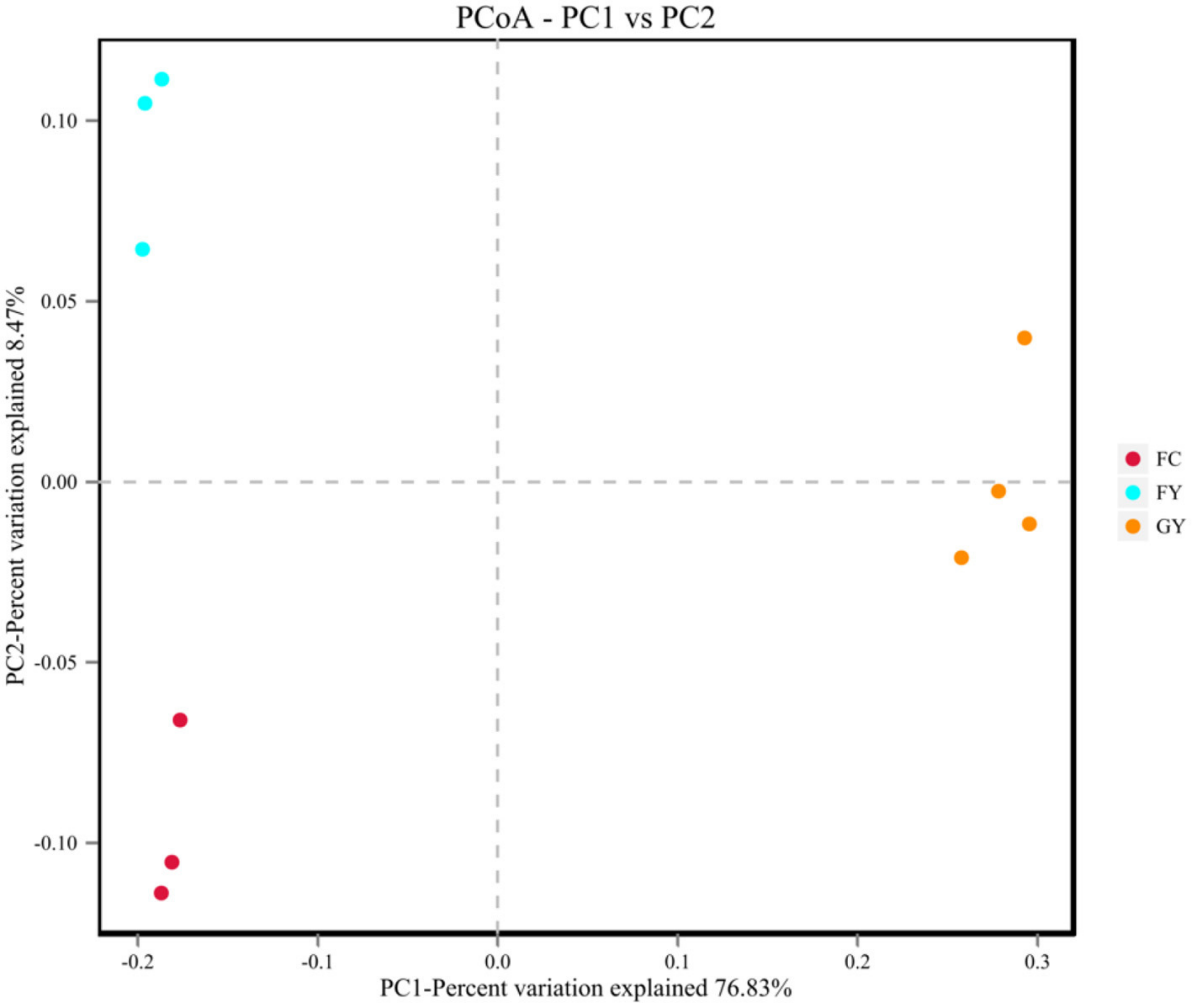
Principal coordinates analysis (PCoA) of rumen bacteria of GYs, FC, and FYs.

A total of 160,598 raw bacterial 16S rRNA gene sequences were obtained after quality control and filtering, and 73,332 valid sequences were analyzed. The ruminal bacteria contained 1,134 OTUs by 16S rRNA gene sequencing. The three groups of bovines shared 675 OTUs of bacteria (Figure 2), while there were 123 non-shared OTUs in GY, 11 OTUs in FY, and 3 OTUs in FC. The ruminal microbial diversity (Shannon index) of GY was greater, but species richness (Chao 1 index) was lesser (*P* < 0.05) than in FY and FC (Figure 3). The rumen microbial community was separated clearly among GY, FY, and FC (Figure 4).

There were 10 bacterial phyla identified in the rumen in the three groups, with Bacteroidetes and Firmicutes being dominant (Table 4). The relative abundance of Bacteroidetes in GY was greater (*P* = 0.03) than in FC, but did not differ from that in FY; whereas, that of Firmicutes did not differ among groups. The relative abundance of Proteobacteria in FC tended to be greater (*P* = 0.09) than in GY or FY, while there was no difference in Kiritimatiellaeota among the three groups. The relative abundances of Fusobacteria and Actinobacteria were lesser (*P* < 0.01) in GY than in FC and FY; *uncultured_bacterium*_k_Bacteria were greater (*P* = 0.02) in FC than in GY and FC; and Synergistetes and Patescibacteria were greater (*P* < 0.05) in GY than in FY and FC. The relative abundance of Chloroflexi was greater (*P* = 0.04) in FY than FC, but not different from that in GY.

**Table 4:** Relative abundance of ruminal bacterial communities at phylum and genus levels of GYs, FC, and FYs.

**Indexes**	**GY**	**FC**	**FY**	**SEM**	***P*-value**
**Phylum, %**					
Bacteroidetes	48.7^b^	29.6^a^	38.6^a^^,^^b^	0.032	0.034
Firmicutes	37.0	35.6	43.2	0.032	0.546
Proteobacteria	2.69	27.1	3.12	0.048	0.089
Fusobacteria	0.02^a^	3.50^b^	3.00^b^	0.006	0.035
Kiritimatiellaeota	2.74	0.62	1.77	0.006	0.231
uncultured_bacterium_k_Bacteria	0.09^a^	0.47^a^	3.97^b^	0.007	0.018
Actinobacteria	1.00^a^	1.62^b^	1.56^b^	0.001	0.038
Synergistetes	2.40^b^	0.08^a^	0.50^a^	0.004	0.026
Patescibacteria	1.99^b^	0.49^a^	0.05^a^	0.003	0.018
Chloroflexi	0.22^ab^	0.13^a^	1.98^b^	0.004	0.043
Others	3.13	0.82	2.24	0.006	0.150
F/B	0.81	1.19	1.13	0.001	0.554
**Genus, %**					
*Prevotella*_1	13.0	14.8	13.1	0.015	0.786
*Succiniclasticum*	6.02	17.4	6.42	0.027	0.150
*Succinivibrionaceae*_UCG-001	0^a^	25.0^b^	0.56^a^^,^^b^	0.049	0.019
*uncultured_bacterium_*f*_Muribaculaceae*	0.70^a^	3.97^a^^,^^b^	17.6^b^	0.028	0.018
*uncultured_bacterium_*f_F082	7.19	2.09	2.50	0.013	0.437
*Christensenellaceae*_R-7_group	3.83^a^^,^^b^	0.60^a^	9.40^b^	0.013	0.018
*Rikenellaceae*_RC9_gut_group	7.71^b^	1.76^a^	1.31^a^	0.011	0.030
*Lactobacillus*	0.08^a^	3.80^b^	3.17^b^	0.006	0.038
*Ruminococcaceae*_NK4A214_group	1.99^a^^,^^b^	0.59^a^	3.70^b^	0.005	0.043
*uncultured_bacterium*_f_*Bacteroidales*_UCG-001	5.03^b^	0.12^a^^,^^b^	0.05^a^	0.009	0.024
Others	50.6^b^	30.0^a^	42.2^b^	0.032	0.026

a,b *Means within a row followed by different lower case letters differ significantly from each other (P < 0.05)*.

*F/B, Firmicutes/ Bacteroidetes*.

There was no difference in the relative abundances of *Prevotella*_1, *Succiniclasticum*, and *uncultured_bacterium*_f_F082 among the three bovine groups; *Succinivibrionaceae*_UCG-001 was greater (*P* = 0.02) in FC than in GY (not detected), but not different than in FY; *uncultured_bacterium*_f_*Muribaculaceae* was greater (*P* = 0.02) in FY than in GY, but not different from that in FC; the *Christensenellaceae*_R-7_ and *Ruminococcaceae*_NK4A214_groups were greater (*P* < 0.01) in FY than in FC, but not different from those in GY. In addition, the *Rikenellaceae*_RC9_gut_group was greater (*P* = 0.03) in GY than in FC and FY; *uncultured_bacterium*_f_*Bacteroidales*_UCG-001 was greater (*P* = 0.02) in GY than in in FY, but not different from that in FC; *Lactobacillus* was lesser (*P* < 0.05) in GY than in FC and FY. More than 50% in GY and 40% in FY of the ruminal bacteria genera were not identified, which was higher (*P* < 0.05) than the 30% in FC (Table 4).

**Figure 5:**
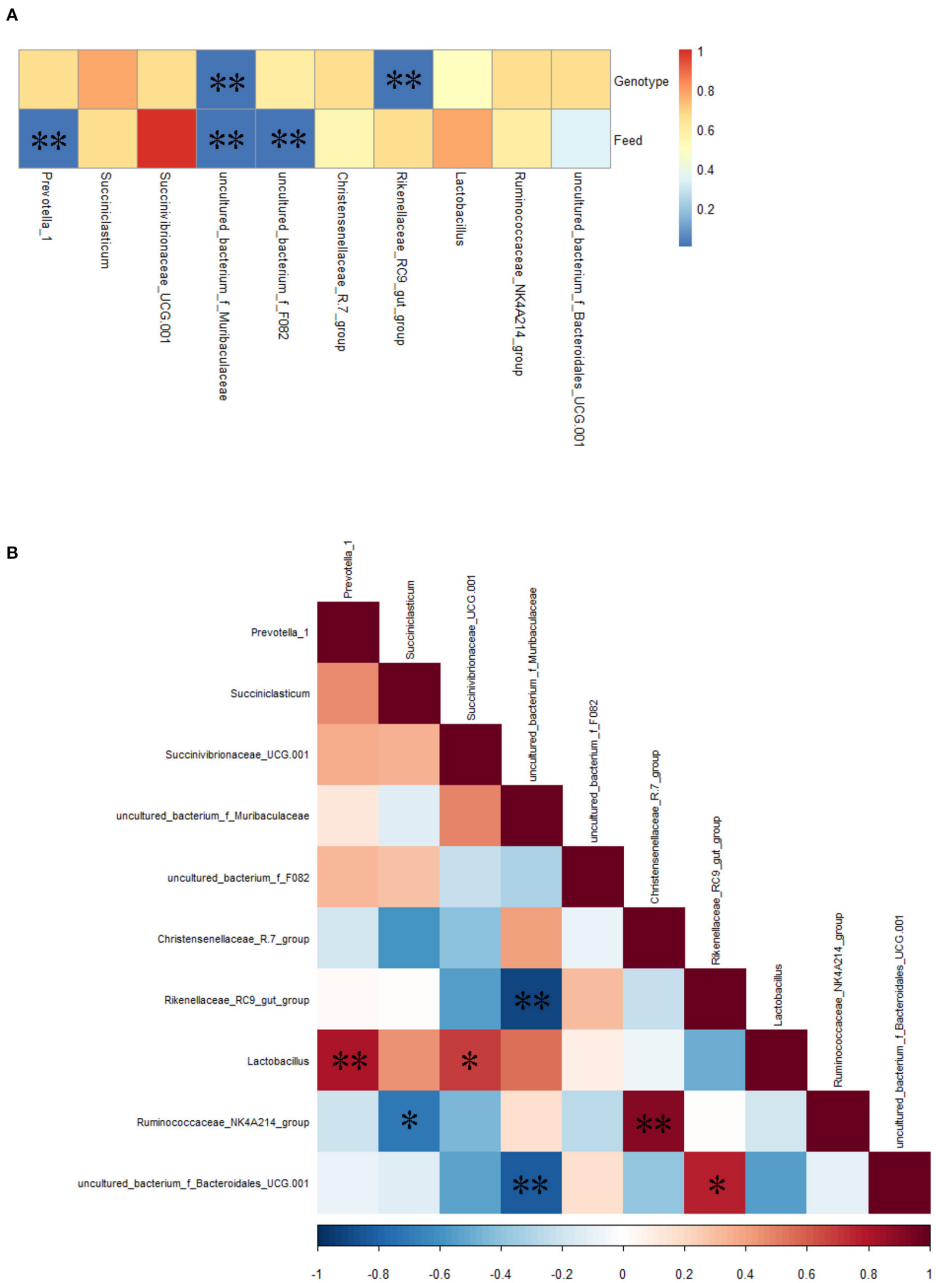
**(A)** Spearman correlations between the top 10 most abundant genera of rumen bacteria and bovine genotype and feed, **(B)** mutual-correlation among the top 10 genera. Color indicates **(A)** *P*-values and **(B)** correlation index (*r*); **(B)** Red represents a positive correlation, and blue represents a negative correlation. *P < 0.05, **P < 0.01.

Spearman correlation analysis showed that the relative abundance of *uncultured_bacterium*_f_*Muribaculaceae* and *Rikenellaceae*_RC9_gut_group was correlated significantly (*P* < 0.01) with bovine genotype (FY vs. FC); the relative abundance of *Prevotella*_1, *uncultured_bacterium*_f_*Muribaculaceae*, and *unculcured_bacterium_*f_F082 was correlated significantly (*P* < 0.01) with feed (GY vs. FY) (Figure 5A). There were significant (*P* < 0.05) positive correlations between *Lactobacillus* and *Prevotella*_1, *Lactobacillus* and *Succinivibrionaceae*_UCG-001, the *Ruminococcaceae*_NK4A214_ and *Christensenellaceae*_R-7_groups, and *uncultured*_*bacterium*_f_*Bacteroidales_*UCG-001 and the *Rikenellaceae*_RC9_gut_group; and negative correlations (*P* < 0.05) between the *Rikenellaceae*_RC9_gut_group and *uncultured_bacterium*_f_*Muribaculaceae*, the *Ruminococcaceae*_NK4A214_group and *Succiniclasticum*, and *uncultured*_*bacterium*_f_*Bacteroidales_*UCG-001 and *uncultured_bacterium*_f_*Muribaculaceae* (Figure 5B).

**Figure 6:**
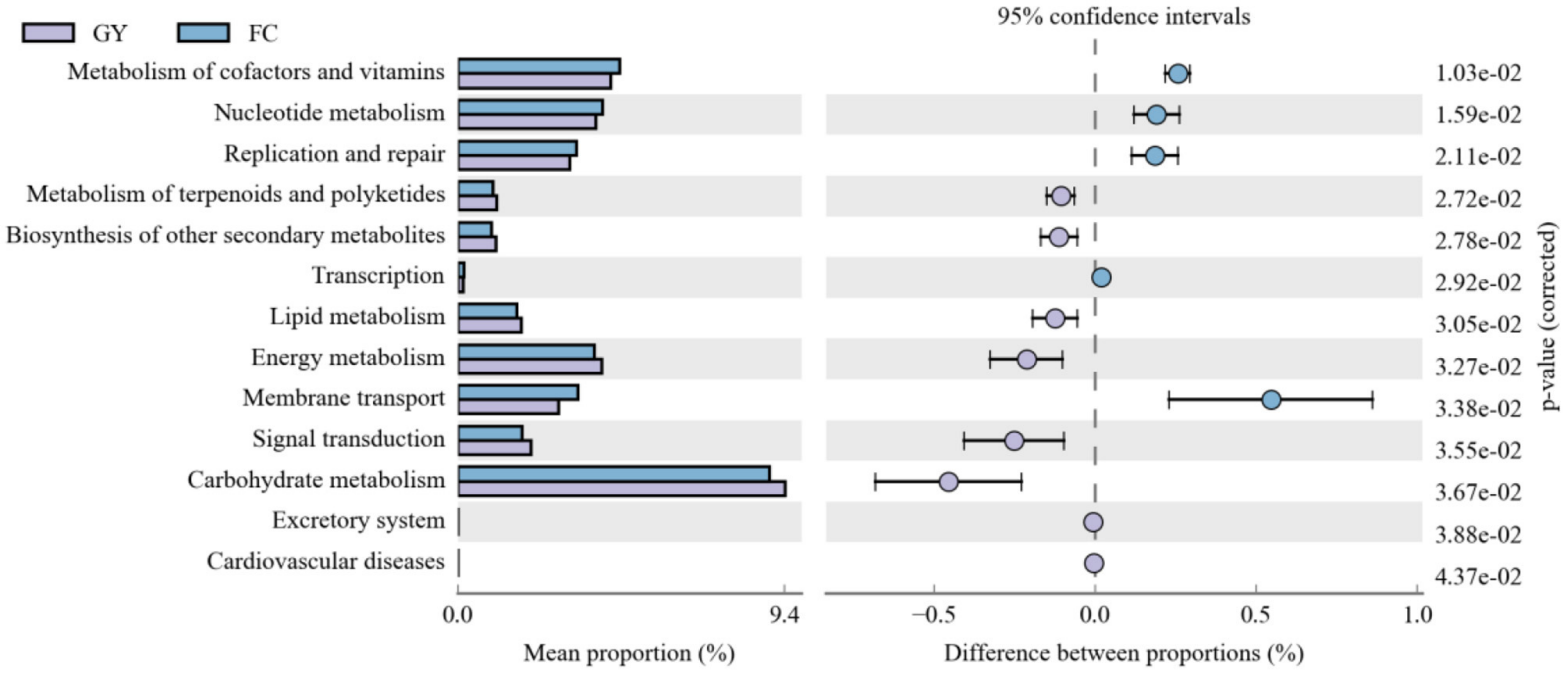
Kyoto Encyclopedia of Genes and Genomes (KEGG) analysis of rumen bacteria between GYs and FC.

Eleven significant rumen microbial metabolic pathways were identified between GY and FC by the Kyoto Encyclopedia of Genes and Genomes (KEGG) analysis (Figure 6). The rumen microbial functions of metabolism of co-factors and vitamins, nucleotide metabolism, replication and repair, transcription, and membrane transport were greater in FC than in GY. However, the metabolism of terpenoids and polyketides, biosynthesis of other secondary metabolites, lipid metabolism, energy metabolism, signal transduction, and carbohydrate metabolism were greater in GY than in FC.

### Meat Quality

The moisture content of meat did not differ among the three bovine groups. The crude protein content was higher (*P* < 0.01), but the fat content was lower (*P* < 0.05), in meat of the two yak groups (GY and FY) than in FC (Table 5).

**Table 5:** Chemical composition of *Longissimus thoracis* from GYs, FC, and FYs.

**Item**	**GY**	**FC**	**FY**	**SEM**	***P***
Water, % fresh meat	67.1	70.5	69.5	1.05	0.380
Crude protein, % DM	67.4^b^	56.6^a^	77.2^b^	4.04	0.009
Crude fat, % DM	18.5^a^	43.2^b^	17.9^a^	5.12	0.038

a,b *Means within a row followed by different lower case letters differ significantly from each other (P < 0.05)*.

Myristic acid (C14:0) and myristoleic acid (C14:1) concentrations were higher (*P* < 0.05) in FC than in GY and FY (Table 6). Concentrations of palmitoleic (C16:1) and heptadecenoic acids (C17:1) tended to be lower (*P* = 0.06) in FY than in GY and FC; whereas the concentration of stearic acid (C18:0) tended to be lower (*P* = 0.06) in FC than in GY and FY. The concentration of oleic acid (C18:1n9c) tended to be higher (*P* = 0.051) in FC than in the two yak groups; whereas the concentrations of linolelaidic acid (C18:2n6t) and linoleic acid (C18:2n6c) were lower (*P* < 0.05) in GY than in FC and FY. The concentration of α-linolenic acid (C18:3n3) was higher (*P* < 0.01) in GY than in FC and FY, the concentration of γ-linolenic acid (C18:3n6) was not detected in GY, and the concentration of tricosanoic acid (C23:0) was not detected in FC. Concentrations of henicosanoic acid (C21:0) and tricosanoic acid (C23:0) tended to be greater (*P* < 0.1) in GY than in FC and FY. Fatty acid cis-11,14,17-eicosatrienoic acid (C20:3n3) was detected only in GY, arachidonic acid (C20:4n6) was detected only in FC, and erucic acid (C22:1n9) was detected in GY and FC but not in FY. Fatty acids cis-13,16-docosadienoic acid (C22:2) and cis-4,7,10,13,16,19-docosahexaenoic acid (C22:6n3) were detected only in GY. There was a tendency (*P* = 0.06) of the total saturated fatty acid (SFA) content to be lower in FC than in GY and FY. The concentration of monounsaturated fatty acids (MUFAs) was higher in FC than in FY, but did not differ from that in GY. There was no significant difference in polyunsaturated fatty acids (PUFAs), medium-chain fatty acids (MCFAs), long-chain fatty acids (LCFAs), PUFA:SFA ratio, n3 fatty acids, antherogenic index (AI), and thrombogenicity index (TI) among the three groups. The concentration of n6 fatty acids was lower in GY than in FY, and the n6:n3 ratio of meat was 2.3 in GY, which was lower (*P* = 0.002) than the 8.1 in FC and 11.4 in FY.

**Table 6:** Fatty acid composition (g/100 g fatty acid methyl) of *Longissimus thoracis* from GYs, FC, and FYs.

**Item**	**GY**	**FC**	**FY**	**SEM**	***P-*value**
C12:0, Lauric acid	0.08	0.06	0.07	0.007	0.435
C13:0, Tridecanoic acid	0.08	0.01	0.09	0.019	0.230
C14:0, Myristic acid	2.41^a^	3.20^b^	1.88^a^	0.198	0.007
C14:1, Myristoleic acid	0.30^a^	0.97^b^	0.22^a^	0.117	0.001
C15:0, Pentadecanoic acid	0.60	0.85	1.47	0.234	0.334
C15:1, cis-10-Pentadecenoic acid	0.11	0.04	0.11	0.017	0.152
C16:0, Palmitic acid	21.43	22.74	21.40	0.527	0.569
C16:1, Palmitoleic acid	4.26	4.73	3.33	0.247	0.055
C17:0, Heptadecanoic acid	1.20	1.14	0.88	0.100	0.433
C17:1, cis-10-Heptadecenoic acid	0.80	0.80	0.61	0.040	0.060
C18:0, Stearic acid	21.76	14.51	22.99	1.621	0.062
C18:1n9t, Elaidic acid	4.63	2.68	4.41	0.497	0.246
C18:1n9c, Oleic acid	36.51	42.29	34.28	1.388	0.051
C18:2n6t, Linolelaidic acid	0.11^a^	0.26^b^	0.21^b^	0.022	<0.001
C18:2n6c, Linoleic acid	1.72^a^	3.90^b^	4.77^b^	0.518	0.010
C18:3n3, α-Linolenic acid	0.67^b^	0.16^a^	0.27^a^	0.087	0.008
C18:3n6, γ-Linolenic acid	–	0.06	0.06	0.011	0.680
C20:0, Arachidic acid	0.30	0.12	0.20	0.045	0.316
C20:1n9, cis-11-Eicosenoic acid	0.02	0.02	0.00	0.006	0.310
C20:2, cis-11,14-Eicosadienoic acid	0.16	0.10	0.11	0.026	0.584
C20:3n6, cis-8,11,14-Eicosatrienoic acid	0.60	0.10	0.31	0.141	0.382
C20:3n3, cis-11,14,17-Eicosatrienoic acid	0.36	–	–	0.098	
C20:4n6, Arachidonic acid	–	0.01	–	0.003	
C20:5n3, cis-5,8,11,14,17-Eicosapentaenoic acid	0.16	0.45	0.24	0.075	0.270
C21:0, Henicosanoic acid	0.54	0.23	0.27	0.065	0.068
C22:0, Behenic acid	0.18	0.16	0.20	0.030	0.915
C22:1n9, Erucic acid	0.02	0.02	0.00	0.006	0.310
C22:2, cis-13,16-Docosadienoic acid	0.02	–	–	0.005	
C22:6n3, cis-4,7,10,13,16,19-Docosahexaenoic acid	0.07	–	–	0.014	
C23:0, Tricosanoic acid	0.61	–	1.24	0.185	0.079
C24:0, Ligoceric acid	0.05	0.04	0.13	0.024	0.314
SFA	48.79	43.66	50.57	1.310	0.083
MUFA	46.57^a^^,^^b^	50.89^b^	42.99^a^	1.281	0.029
PUFA	4.63	5.45	6.44	0.476	0.325
MCFA	31.26	34.55	30.05	0.874	0.101
LCFA	65.45	69.95	68.74	0.874	0.101
PUFA/SFA	0.10	0.12	0.13	0.011	0.441
n3	1.19	0.61	0.51	0.154	0.121
n6	2.46^a^	4.33^a^^,^^b^	5.36^b^	0.519	0.030
n6/n3	2.30^a^	8.10^b^	11.42^b^	1.426	0.002
AI	0.61	0.64	0.59	0.020	0.728
TI	1.38	1.81	1.62	0.094	0.210

a,b *Means within a row followed by different lower case letters differ significantly from each other (P < 0.05)*.

*TFAs, total fatty acids; SFAs, saturated fatty acids; MUFAs, monounsaturated fatty acids; PUFAs, polyunsaturated fatty acids; MCFAs, medium-chain fatty acids (C11:0–C17:0); LCFAs, long-chain fatty acids (C18:0–C24:0); AI means atherogenic index = (C12:0+4 × C14:0 + C16:0)/(MUFAs+ PUFAs); TI means thrombogenicity index = (C14:0 + C16:0 + C18:0)/[(0.5 × MUFAs) + (0.5 × n6 PUFAs) + (3 × n3 PUFAs) + n3/n6 PUFAs]*.

“–” *means not detected*.

The meat of GY contained higher (*P* < 0.05) concentrations of isoleucine, tryptophan, tyrosine, leucine, and essential amino acids (EAAs) than the meat of FC, and the concentrations in FY did not differ from those in either group. Glutamic acid concentration in FC was lower than in GY and FY (*P* = 0.03); whereas the concentrations of methionine (*P* = 0.008) and phenylalanine (*P* = 0.001) were higher in GY than in FY and FC. The ratios of EAA acids to total amino acids (TAAs) and to non-essential amino acids (NEAAs) were higher (*P* < 0.001) in GY than in FY and FC, and in FY than in FC (Table 7).

**Table 7:** Amino acid composition (% DM) of *Longissimus thoracis* from GYs, FC, and FYs.

**Item**	**GY**	**FC**	**FY**	**SEM**	***P*-value**
Glycine	1.02	1.80	1.20	0.179	0.187
Alanine	7.69	7.89	8.19	0.289	0.816
Serine	0.92	0.93	0.73	0.080	0.597
Proline	1.51	1.13	1.52	0.103	0.262
Valine	2.01	1.24	1.63	0.141	0.055
Threonine	1.71	1.36	1.66	0.101	0.375
Cystine	0.05	0.07	0.05	0.005	0.383
Leucine	2.23^b^	1.16^a^	1.71^a^^,^^b^	0.177	0.015
Isoleucine	1.34^b^	0.63^a^	0.96^a^^,^^b^	0.119	0.019
Glutamic acid	2.07^a^	7.25^b^	1.90^a^	1.017	0.033
Methionine	1.12^b^	0.42^a^	0.78^a^	0.125	0.008
Histidine	0.54	0.51	0.41	0.044	0.531
Phenylalanine	1.48^c^	0.59^a^	1.12^b^	0.133	0.001
Arginine	2.56	1.64	2.10	0.263	0.394
Tryptophan	0.41^b^	0.23^a^	0.35^a^^,^^b^	0.030	0.022
Lysine	2.63	2.21	2.14	0.215	0.637
Tyrosine	0.95^b^	0.40^a^	0.66^a^^,^^b^	0.093	0.022
TAA	30.30	29.47	27.12	1.793	0.799
EAA	12.99^b^	7.85^a^	10.35^a^^,^^b^	0.927	0.043
EAA/TAA	0.43^c^	0.27^a^	0.38^b^	0.024	<0.001
EAA/NEAA	0.76^c^	0.36^a^	0.62^b^	0.058	<0.001

a−c *Means within a row followed by different lower case letters differ significantly from each other (P < 0.05)*.

*TAAs, total amino acids; EAAs, essential amino acids; NEAA, non-essential amino acids*.

## Discussion

### Rumen Morphology

Concentrate feed has been reported to stimulate the proliferation and growth of rumen epithelium (14, 15). For example, goats supplemented with dietary concentrate feed increased the length of rumen papillae when compared with grazing goats (16). This could explain the increased rumen papillae length of the two groups consuming concentrate in this study, especially for the feedlot yaks (FYs), when compared with the grazing yaks (GYs). These results also indicated that the yak rumen can adapt to a high-grain diet. High-grain diet can enhance the production of short-chain fatty acids (volatile fatty acids, VFAs) in the rumen, which are absorbed mainly by the rumen epithelium (17). The rumen epithelium absorbs 50–80% of VFAs, and depends mainly on the papillae surface area (3, 18). Therefore, the higher papillae surface area of FY facilitated the absorption of VFAs and nutrients (6) when compared with GY. It was also reported that the *stratum corneum*, which is formed in layers, can have four times the number of layers in a ruminant consuming a high concentrate diet than a high forage diet (19, 20). However, in this study, in contrast to these reports, the GY group had a thicker *stratum corneum* than the two feedlot bovines (FY and FC). The *stratum corneum* is in direct contact with the rumen milieu, and was observed to slough off throughout the epithelial surface when feeding high grain diet (15). Further research is required to explain this difference in *stratum corneum* thickness among groups and between feeds.

### Ruminal Microbiota and Fermentation

Several methods have been employed to collect rumen samples for determining relative abundances of microbiota, namely, *via* oro-ruminal tube (21–24) and ruminal fistula (25, 26), and collection from a slaughtered animal (5, 27). The contents are often strained through cheesecloth and snap-frozen at −80°C until analyzed. It has been reported that the solid part of rumen contents contains ~70% of microbial mass, the liquid part ~25%, and the rumen epithelium and protozoa approximately 5% (28). Comparative studies have shown that there are differences in the relative abundances of bacteria among the methods of collection and among rumen samples (fluid, solid, or whole contents) analyzed (29, 30). For example, in the study of Cunha et al. (30) on Holstein cows, “mean relative abundance of Fibrobacteres was greater in solid and mixed liquid-solid than in liquid and oral samples,” whereas “no difference in mean relative abundance was detected in the 30 most prevalent genera among sample types.” Hendersen et al. (29), in their study on cows and sheep, stated that “the liquid fraction appeared to contain a higher relative abundance of the family *Prevotellaceae* (1.4-fold increase, 25.1–36.1%) and a lower abundance of the family *Lachnospiraceae* (1.6-fold decrease, 12.7–20.8%) when compared with total (and solid) rumen sample fraction. These findings generally agree with those of others” (three references are provided). Therefore, this study indicates that the same relation in relative abundance of bacteria exists across diets when comparing the different rumen samples, and that comparisons among studies on rumen bacteria cannot be made unless the same method of measurement is used. However, it also indicates that comparisons within a study can be made if the same method of measurement is used for all animals. In this study, the handling of rumen contents and preparation of the samples were performed in the same manner for all animals and were performed by the same person and, consequently, comparisons among the three bovine groups could be made.

The ruminal bacteria OTU data indicated that diet affected the bacterial community, more so than genotype. FY had more common communities with FC than with GY by more than a 4-fold difference. In addition, GY had 11-fold and 41-fold more unique OTUs when compared with FY and FC, respectively, which also explained the greater bacterial community diversity in GY than in FY and FC. The total OTUs of GY, which was lower than that of FY and FC, was also reflected by the Chao1 index. The greater diversity was a consequence of the high number of plant species in the natural pasture consumed by GY when compared with FY and FC. Natural forage diets provide a greater range of carbohydrate substrates (e.g., cellulose, semi-cellulose) and enhance the growth of microorganisms (31). In addition, high grain diets reduced rumen microbial richness and diversity (4, 32), which is related to higher feed efficiency (33). Nutrient-rich diets do not supply optimal fermenting substrate for all rumen microbes, especially for fiber-digesting bacteria (34, 35). The enhanced ruminal diversity with forage, when compared with high starch and lipid diets, is a consequence of the long-term co-evolution of microbes and yaks grazing on Alpine pasture. The higher Shannon index and lower Chao1 index of GY, compared with those of FY and FC, indicated greater evenness of OTUs in the GY group. Forage consumed by GY could be the reason for the high OTU evenness, as forages displayed more uniform OTU distributions than corn in denaturing gradient gel electrophoresis (DGGE) libraries (36).

Bacteroidetes and Firmicutes were the dominant phyla in the three bovine groups, which is consistent with previous studies (37–39). It was reported that a high concentrate diet decreased the ruminal abundance of Bacteroidetes, and increased the abundance of Firmicutes in cattle (31, 40, 41). This was also observed, at least partially, in this study, where the ruminal abundance of Bacteroidetes was lower in FC than in GY. The abundance in FY was intermediate between FC and GY but did not differ from that in either group. Firmicutes were not affected by the concentrate intake in this study, which could be, at least in part, because of the 18-h post-feeding sampling time. Previous studies have reported that Firmicutes were highest just before feeding and lowest ~12 h post feeding (42). It was reported that the ratio of F:B increased with a high energy diet (5, 43). This trend occurred in this study, albeit the differences were not significant, as GY had a ratio of 0.81, whereas FC had a ratio of 1.19, and FY had a ratio of 1.13. In this study, the relative abundance of Proteobacteria, which has little, if any, and cellulolytic activity (44) tended to be greater in FC than in GY and FY. Sheep breeds were reported to affect the abundance of Proteobacteria, which consists mostly of pathogenic bacteria (45). Therefore, the low abundance of Proteobacteria in the two yak groups indicates that this bacterial phylum is affected by genotype, as it was in sheep, and that yaks have a stronger ability to resist pathogens than cattle. The relative abundance of ruminal Fusobacteria was greater in FC and FY than in GY. Fusobacterial infections can cause liver abscesses in cattle (46), which indicates that high-concentrate feeding has the risk of causing liver damage. Studies demonstrated that a dietary shift from concentrate to pasture increased the abundance of ruminal Actinobacteria in sheep (23), but that its role is still unknown (47). High protein diets increased the relative abundance of ruminal Synergistetes that degrade amino acids rather than carbohydrates (48, 49). The high digestible carbohydrate proportion in the diet of FC and FY could be the reason for the inhibition of growth of Synergistetes. Few reports are available on ruminal Patescibacteria; however, the current results demonstrated that high-concentrate diets reduced the relative abundance of this phylum. In this study, the relative abundance of Chloroflexi increased with concentrate intake, as was reported earlier (50), but the function of this phylum is still uncertain.

*Prevotella*_1 and *Succiniclasticum* are the dominant ruminal genera in this study, as has been reported earlier in bovines (51). *Prevotella*_1, which uses mainly proteins as a substrate for growth (52), was correlated significantly with feed; whereas *Succiniclasticum* specializes in fermenting succinate to produce propionate (53). *Ruminococcaceae*, which was observed in different ovine breeds (54, 55), is a cellulolytic family of bacteria that plays a role in biohydrogenation (56). Hydrogen can be incorporated into succinate to increase *Christensenellaceae*, which is involved in carbohydrate digestion, and increases at the mid-fattening period of cattle (57, 58). However, in this study, the *Ruminococcaceae*_NK4A214_group was not correlated with genotype. Among genera, the *Ruminococcaceae*_NK4A214_group was correlated positively with the *Christensenellaceae*-R-7_group but negatively with *Succiniclasticum*. The competition to use succinate could explain the latter negative correlation. The higher relative abundance of the *Christensenellaceae*-R-7_group in FY than in FC was related with the fattening period of yaks.

*Succinivibrionaceae*_UCG-001 was a dominant genus in FC, present in FY but not detected in GY. It was reported that ruminal *Succinivibrionaceae* reduced methane emission (59), as occurs with a high-concentrate diet (60). However, FY had a low relative abundance of *Succinivibrionaceae*_UCG-001, which suggests that, presumably, a longer time period was required for colonization of the bacteria. The proliferation of *Lactobacillus* contributes to rumen lactate production when fed with a high-concentrate diet (61), which could be the reason for the high relative abundance in FC and FY when compared with GY. Concentrate promoted the relative abundance of both *Succinivibrionaceae*_UCG-001 and *Lactobacillus*, which also showed a positive correlation between them.

Rumen *Muribaculaceae* was correlated positively with feed nutrient digestibility in yaks (62). The concentrate intake in FY explained the higher relative abundance of *uncultured _bacterium_f_Muribaculaceae* in these yaks than in grazing yaks. The *Rikenellaceae*_RC9_gut_group degrades cellulose and hemicellulose (63, 64), while *Bacteroidales* enhances fiber digestion and is more abundant in high-forage than low-forage diets (65). Therefore, the high fiber content in forage could explain the increased growth of the *Rikenellaceae*_RC9_gut_group and *uncultured_bacterium_f_Bacteroidales*_UCG-001 in GY. *Bacteroidales* promotes ruminal acetate production (31), which was consistent with the higher acetate concentration in GY than in FC. The high forage fiber increased the abundance of the *Rikenellaceae_RC9_*gut_group and *uncultured_bacterium_f_Bacteroidales_*UCG-001, but decreased the abundance of *uncultured -_bacterium_*f*_Muribaculaceae*. The *uncultured _bacterium_f_Muribaculaceae* and the *Rikenellaceae*_RC9_gut_group were correlated significantly with genotype (yaks vs. cattle) in this study. Except for the top 10 genera, the higher relative abundances of other ruminal genera in the yak groups (GY and FY) than in FC indicate the greater rumen microbial diversity in yaks than in cattle.

Concentrations of ruminal total VFAs and acetate were greater in the two yak groups than in FC, while propionate and butyrate displayed the same pattern albeit were not significantly higher. This would indicate that yaks are more efficient in producing VFAs than cattle, that is, VFA production is dependent mainly on genotype and less so on dietary intake. This premise was supported in the study of Zhou et al., who reported that yaks produced greater concentrations of ruminal total VFAs than Qaidam Yellow cattle when consuming the same diet (66).

The KEGG data revealed that the rumen microbiota from feedlot-fattened cattle (FC) had an enhanced capacity to influence the metabolism of co-factors and vitamins, for nucleotide metabolism, replication and repair, transcription, and membrane transport. These metabolite changes indicate that the rumen microbiota in the FC group exhibited relatively high fluctuations and enhanced turnover rates (32), which were consistent with the reduced diversity and richness in FC when compared with GY.

Compared with FC, GY displayed enhanced metabolism of terpenoids and polyketides, and biosynthesis of other secondary metabolites. These responses were due to the large variety of secondary metabolites, such as polysaccharides, flavonoids and saponins, provided by natural pasture on the Qinghai-Tibetan Plateau (67). The metabolism of lipids, carbohydrates, and energy, all related with feed energy utilization and storage, was enhanced in grazing yaks when compared with the feedlot cattle in this study. Through long-term adaptations to energy stress, yaks have evolved traits of slow fat mobilization and strategies to conserve energy in the harsh alpine environment (68).

### Fatty Acids and Meat Quality

Meat water content reflects meat texture, tenderness, and juiciness (69). The proportion of dietary forage intake did not affect meat water content in steers (4), which is in agreement with this study, as there was no difference among the three groups. The meat of FC had lower crude protein and higher fat contents than that of both yak groups, which did not differ between them, and which is in agreement with the study of Zi et al. (70). This indicates that genotype was the main factor affecting these components, as was stated in an earlier study (71), and that feed had a lesser effect, at least in yaks.

Microbial activity in the rumen is responsible for the hydrolysis of esterified plant lipids and liberation of unsaturated fatty acids, which determines the fatty acid composition of the meat (72). However, diet plays an important role in determining the tissue fatty acid content (73, 74). Myristic acid (C14:0) is regarded as an undesirable fatty acid in food because of its linkage with high blood cholesterol level in humans (75). However, it was reported that C14:0 could enhance intramuscular fat deposition in pork and increase the accumulation of myristic and myristoleic acids (C14:1) in meat tissues, which may have beneficial effects on human health (76). The higher concentrations of C14:0 and C14:1 in FC than in both yak groups could be due mainly to the effect of genotype, as Simmental was found to have a higher carcass C14:1 concentration than Red Angus (77). Palmitoleic acid (C16:1) and oleic acid (C18:1) were converted from palmitic acid (C16:0) and stearic acid (C18:0) by delta-9 desaturase (78). The tendency of higher C18:1n9c and lower C18:0 in FC than in the two yak groups indicates the high activity of delta-9 desaturase and high fat anabolism (79), which is consistent with the higher fat content in FC. It is also consistent with the report of Lobo et al. that genotype plays an important role in body fat anabolism (80). Lower concentrations of linoleic and linolelaidic acids (C18:2n6c and n6t) were observed in the meat of GY than in FC and FY, which was due to the lower content of C18:2 in grass than in concentrate feed (81), as C18:2n6c in meat is derived wholly from dietary intake (82). High levels of C18:2 can have adverse effect on odors and flavors of beef (83). The higher concentration of α-linoleic acid (C18:3n3) in forage than in concentrate feed is the reason for the higher concentration in the meat of GY than in the meat of FC and FY (84, 85), with no effect because of genotype (79). High ingestion of C18:3n3 could result in the increase of DHA (C22:6n3) in meat, which is the elongation product of C18:3n3 (74, 85). This can explain why DHA in meat was detected only in GY in this study. DHA has been reported to possess many healthy properties for humans, such as anti-obesity and improving cognitive function and neuronal development (86).

Particular attention has been paid to n-3 PUFAs, as they are reputed to possess the ability to prevent and treat cancer, coronary artery disease, hypertension, diabetes, and inflammatory and autoimmune disorders (87, 88). Food with a high n6:n3 ratio, however, can cause a number of diseases, such as cardiovascular problems, cancer, and inflammatory and autoimmune disorders (89). The high proportion of α-linoleic acid (n3 fatty acid) in GY led to a lower n6:n3 ratio (2.3) in these yaks than in FC (8.1) and FY (11.4). Most foods have am n6:n3 ratio ranging between 10:1 and 30:1 (88, 90); however, a ratio of <4 was recommended by the British Department of Health (91) to be healthy for humans, and only GY meat met this criterion. Consequently, from the point of DHA content and n6:n3 ratio, grazing yaks provide healthier meat than feedlot-fattened livestock.

Eicosatrienoic acid (C20:3n3) was detected only in GY, and arachidonic acid (C20:4n6) was detected only in FC, which indicates that grazing animals increased n3 fatty acid deposition in meat to a greater extent than feedlot animals fattened on a high concentrate diet. Furthermore, the intake of C18:3n3 increased the contents of n3 PUFAs and decreased n6 PUFAs in the meat of lambs (92), which also explains the lower n6:n3 ratio in GY than in FY and FC. Tricosanoic acid (C23:0), which is involved in the synthesis of ceramide and reduces the risk of diabetes (93, 94), was not detected in FC, but was present in the yaks (GY and FY). The tendency of lower saturated fatty acids (SFAs) in FC than in the two yak groups was attributed mainly to C18:0, which amounted to 30–45% of SFAs. Other studies found no difference in SFAs between confined and grazing cattle meat (95, 96). The higher total concentrations of monounsaturated fatty acids (MUFAs) in FC than in FY was related mainly to oleic acid. The insignificance of MUFAs between GY and FY indicates that feed did not affect the delt-9 desaturase activity to convert SFAs in yaks (97). No difference in PUFA and PUFA:SFA ratio was observed among the three bovine groups in this study. The PUFA:SFA ratios were all below the ratio (0.4) recommended by the World Health Organization (98), which is consistent with the reported values for cattle (~0.1) (54, 99), but lower than values reported for yaks (0.4–0.6) (100) and bison (0.4–0.6) (101). The atherogenic index (AI) and thrombogenicity index (TI) are related to the incidence of coronary heart disease (80, 102); there was no difference in these indices among GY, FC, and FY in this study.

The amino acids in meat affect the taste, at least, of beef (103). Glutamic acid is regarded as an important amino acid that adds flavor in meat (104), and FC had a higher content of glutamic acid than GY and FY. Other amino acids that add flavor to the meat, namely, glycine, alanine, and proline (105), did not differ among the three groups. The yak meat (GY and FY) had higher concentrations of phenylalanine than FC, and GY had higher concentrations than FC and FY of the essential or semi-essential amino acids for humans, namely, leucine, isoleucine, methionine, tryptophan, and tyrosine. According to FAO/WHO (106), healthy food composition should have an EAA:TAA ratio of at least 0.4 and an EAA:NEAA ratio of at least 0.6. Yak meat (GY and FY) obtained these ratios, with GY higher than FY, but FC did not.

## Conclusions

Yaks fattened in a feedlot (FY) had a greater rumen papillae surface area and ruminal microbial diversity, but lesser microbial richness when compared with grazing yaks (GY) and cattle fattened in a feedlot (FC). Both the grazing and feedlot-fattened yaks had lower abundance of ruminal Proteobacteria and higher concentrations of volatile fatty acids than the feedlot-fattened cattle. The yaks, both grazing and feedlot-fattened, produced greater concentrations of ruminal VFAs and had meat with higher protein and lower fat contents than cattle. Meat from the grazing yaks had greater α-linolenic acid (C18:3n3) and lesser linolelaidic acid (C18:2n6t) concentrations, and n6:n3 ratios of fatty acids (<4) than the meat from feedlot-fattened meat (yak or Simmental). Yak meat had healthier ratios of EAA:TAA and EAA:NEAA than cattle meat, but the cattle meat had greater concentrations of flavor amino acids than the meat of the yaks.

## Data Availability Statement

The datasets presented in this study can be found in online repositories. The names of the repository/repositories and accession number(s) can be found at: https://www.ncbi.nlm.nih.gov/sra/?term=PRJNA761282.

## Ethics Statement

The animal study was reviewed and approved by Animal Care and Use Committee of Lanzhou University.

## Author Contributions

LD designed the experiment. CH, CJ, and CM conducted the experiment. CH, LD, and AD wrote and revised the manuscript. BL and DL collected the meat samples. CH did the data analysis. All authors reviewed the manuscript.

## Funding

This research was supported by Qinghai Provincial Science and Technology Major Project (2018-NK-A2), Platform of Adaptive Management on Alpine Grassland-livestock System (2020-ZJ-T07), STS grant from the Chinese Academy of Sciences (KFJ-STS-QYZD-113), Qinghai Key Research and Development Project (2017-NK-114), Key Lab Project of Qinghai Province (2013-Z-Y03), and China Agriculture Research System: National Modern Agriculture (Beef and Yak) Industrial Technology System Project (CARS-37).

## Conflict of Interest

BL is employed by Gansu Devotion Biotechnology Co. Ltd. DL is employed by Qinghai Qilian Yida Meat Co. Ltd. The remaining authors declare that the research was conducted in the absence of any commercial or financial relationships that could be construed as a potential conflict of interest.

## Publisher's Note

All claims expressed in this article are solely those of the authors and do not necessarily represent those of their affiliated organizations, or those of the publisher, the editors and the reviewers. Any product that may be evaluated in this article, or claim that may be made by its manufacturer, is not guaranteed or endorsed by the publisher.
